# Vegetation change in response to climate factors and human activities on the Mongolian Plateau

**DOI:** 10.7717/peerj.7735

**Published:** 2019-09-30

**Authors:** Meng Meng, Ni Huang, Mingquan Wu, Jie Pei, Jian Wang, Zheng Niu

**Affiliations:** 1 The State Key Laboratory of Remote Sensing Science, Institute of Remote Sensing and Digital Earth, Chinese Academy of Sciences, Beijing, China; 2 University of Chinese Academy of Sciences, Beijing, China

**Keywords:** Climate change, The mongolian plateau, GIMMS 3g, Correlation analysis, Residual trend analysis

## Abstract

**Background:**

Vegetation in the Mongolian Plateau is very sensitive to climate change, which has a significant impact on the regulation of terrestrial carbon cycle.

**Methods:**

We analyzed spatio-temporal changes of both growing season and the seasonal Normalized Difference Vegetation Index (NDVI) using simple linear trend analysis. Besides, correlation analysis was applied to explore the climate factors’ effects on vegetation growth at temporal and spatial scale. Potential effects of human factors on vegetation growth were also explored by residual trend analysis.

**Results:**

The results indicated that vegetation growth showed a greening trend in the Mongolian Plateau over the past 30 years. At the temporal scale, the growing season NDVI showed an insignificant increasing trend (at a rate of 0.0003 yr^−1^). At the spatial scale, a large region (53.8% of the whole Mongolian Plateau) with an increasing growing season NDVI, was primarily located in the southern and northern parts of the plateau. The correlation analysis suggested that temperature and precipitation were the main limiting factors that affected vegetation growth in spring and the growing season, respectively. The residual trend analysis showed that human activities primarily stimulated the growth of grasslands and shrublands, while croplands displayed a decreasing trend due to human disturbances, implying that anthropogenic factors may lead to croplands abandonment in favor of grasslands restoration. Our results provided detailed spatial and temporal changes of vegetation growth, and explored how climate and human factors affected vegetation growth, which may offer baseline data and scientific suggestions for local land and resources management, and facilitate the sustainable development of the terrestrial ecosystems.

## Introduction

The fifth assessment report of the United Nations Intergovernmental Panel on Climate Change (IPCC) noted that the climate of Northern Hemisphere has become growing warmer over the past 30 years (Allen et al., 2014). Many aspects of vegetation have been influenced by such a sudden warming, such as vegetation diversity, biomass, productivity, phenology, and vegetation fractions (Liu et al., 2013; Liu et al., 2017; Tsvetsinskaya et al., 2003). These changes in vegetation may also incorporate feedback into climate change (Wu, 2016). Therefore, there has been increasing attention given to explore global climate change and its impacts on vegetation dynamics (White, Thornton & Running, 1997; Zhu et al., 2016).

The utilization of remotely sensed satellite data to monitor vegetation growth has improved our understanding of vegetation dynamics. The Normalized Difference Vegetation Index (NDVI) is considered as an optimal indicator of vegetation growth (Piao et al., 2015). In recent years, time-series NDVI datasets have been adopted to monitor vegetation dynamics at different spatio-temporal scales. For instance, Chakraborty et al. (2018) evaluated the spatial distribution of forest seasonal greenness in India through applying the Moderate-resolution Imaging Spectroradiometer (MODIS) NDVI datasets during 2001–2014. Fan et al. (2018) investigated vegetation changes of Poyang Lake basin, China using the MODIS NDVI data from 2001 to 2015. Kundu et al. (2018) explored vegetation cover changes of Bundelkhand Region (India) by applying the Systeme Probatoire d’Observation de la Terre VEGETATION (SPOT-VGT) NDVI datasets during 1998–2013. Previous research demonstrated that the MODIS NDVI and the SPOT-VGT NDVI datasets all had an advantage of higher spatial resolution, but time series of these two datasets were relatively short. The Global Inventory Modeling and Mapping Studies (GIMMS) NDVI datasets have been proven to be the longest remotely sensed time series data for the exploration of long-term vegetation growth dynamics, which have been widely used to monitor vegetation changes on large scales (Baniya et al., 2018; Liu et al., 2017; Peng et al., 2011).

The Mongolian Plateau is a vast inland region and has been shown to be a key area that is sensitive to climate change (Zhang et al., 2009; Chuai et al., 2013). In recent years, the vegetation has been threatened by climate and human factors (Eckert et al., 2015; Zhou, Yamaguchi & Arjasakusuma, 2017). Many previous studies analyzed the vegetation changes in Mongolian Plateau through using long-term satellite remote sensing data, but have not reached consensus on its spatio-temporal changes as well as its responses to climate change. For example, Xia et al. (2015) found that vegetation growth showed an insignificant increasing trend over the entire plateau during 1982–2011, and climate factors (i.e., precipitation and air temperature) were the two most important variables affecting vegetation growth. Bao et al. (2014) and Chuai et al. (2013) pointed out that the effects of climate factors on vegetation growth varied with time scale, and growing season and summer vegetation growth were primarily controlled by precipitation, while temperature was the major limiting factor for spring vegetation growth from the 1980s to the late 1990s. The difference in time periods may partly explain these inconsistences and it is necessary to reanalyze vegetation change in the Mongolian Plateau at various time scales (i.e., annual, spring and summer). In addition, human activities were another important factor affecting vegetation growth (Liu et al., 2015; Xin, Xu & Zheng, 2008). However, there have been rather limited literature on analyzing vegetation changes induced by human activities in the Mongolian Plateau (Zhou, Yamaguchi & Arjasakusuma, 2017). Comprehensively considering the effects of climate factors and human activities is of great significance for improving our understanding of the vegetation changes in the Mongolian Plateau.

Based on the GIMMS NDVI 3g and monthly gridded meteorological datasets from 1982 to 2015, we first analyzed the spatio-temporal vegetation changes in growing season, spring, summer, and autumn by simple linear trend analysis. We then explored the responses of vegetation dynamics to climate factors for different plant types using correlation analysis. Besides, the potential effects of human activities on vegetation changes were also investigated using residual trend analysis. The overall questions we addressed are how climate and anthropogenic factors affect the interannual and seasonal NDVI changes over the whole plateau over a longer period of time (1982–2015), and how such impacts vary in different vegetation types over the plateau. It is expected that these findings could provide a perspective into the most sensitive regions to environmental changes within the Mongolian Plateau, and support the sustainable land management and development of terrestrial ecosystems.

## Materials & Methods

### Study area

**Figure 1:**
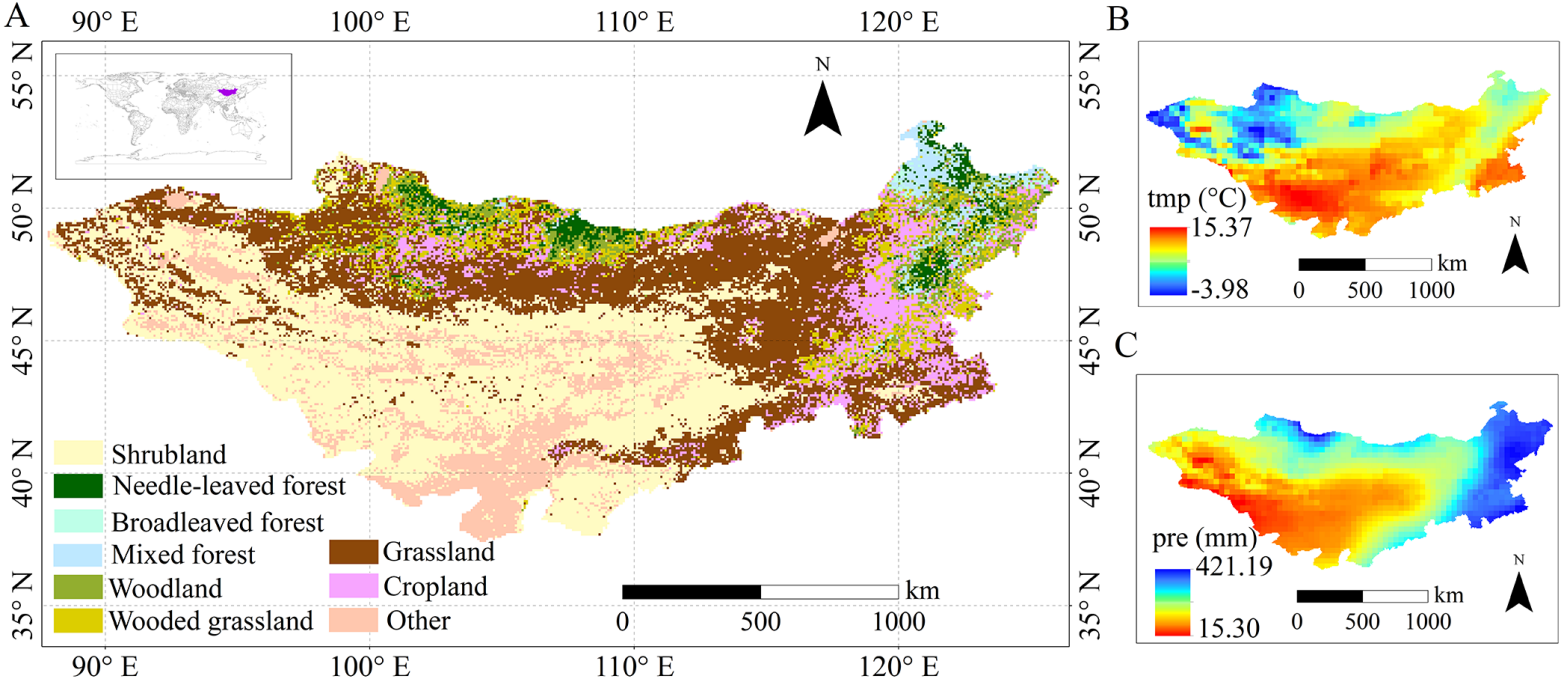
The spatial distribution of (A) vegetation types in the Mongolian Plateau; (B) growing season mean air temperature (tmp); and (C) growing season average precipitation (pre).

Our study region (87°43′–126°04′E, 37°22′–53°20′N) includes all of Mongolia and the Inner Mongolia Autonomous Region (IMAR) with an area of approximately 2,700,000 km^2^. The Mongolian Plateau has a typically temperate continental monsoon climate. The annual mean temperature ranges from 1.5 °C or lower in the northern mountains to 16 °C or higher in the southwestern Gobi Desert (Fig. 1B). The annual average precipitation is less than 200 mm in most regions, but it may reach 400 mm or higher in the northeastern forest regions (Fig. 1C). The vegetation types across the plateau also show spatial differences due to the physical distribution of the climate (Fig. 1A).

### NDVI data

The NDVI can be used as a proxy for terrestrial vegetation growth due to its close correlation with vegetation biomass, vegetation productivity, and fractional vegetation coverage (Peng et al., 2011; Defries & Townshend, 1994). We applied the third generation GIMMS biweekly NDVI 3g.v1 datasets with a spatial resolution of 1/12° from 1982–2015, which were acquired from (https://ecocast.arc.nasa.gov/data/pub/gimms/3g.v1). The Maximum Value Composite (MVC) method was used to obtain the monthly NDVI data (Peng et al., 2011), as shown in the following formula.

(1) \begin{eqnarray*}MNDV{I}_{i}=\mathrm{Max}(NDV{I}_{1},\,NDV{I}_{2})\end{eqnarray*}

where *i* is the series number representing the 4th to 10th month; *MNDVIi* is the NDVI for the *i* th month; and *NDVI*_1_ and *NDVI*_2_ are the NDVI for the first and second halves of the *i* th month, respectively.

Growing season NDVI for each year is defined as the NDVI from April to October (Piao et al., 2011b). Furthermore, we defined seasonal NDVI as follows: spring was from April and May; summer was from June to August; autumn was from September and October (Peng et al., 2011; Piao et al., 2011b). Moreover, we defined non-vegetation regions as pixels that had an average NDVI of less than 0.1 (Piao et al., 2011a).

### Meteorological data

In this study, precipitation and temperature were adopted as the meteorological data. The monthly gridded Climatic Research Unit Timeseries version 4.01 (CRU TS 4.01) meteorological data were obtained from the Climatic Research Unit, University of East Anglia (http://data.ceda.ac.uk/badc/cru/data/cru_ts_4.01/), with a spatial resolution of 0.5° (Baniya et al., 2018; Zhang et al., 2013). Accumulated precipitation and average air temperature for growing season and three seasons were calculated for the time span of 1982 to 2015, respectively. To match the spatial resolution of GIMMS NDVI3g.v1 data, we used the nearest-neighbor interpolation method to resample the climate data to 1/12°.

### Land cover data

We utilized the Advanced Very High Resolution Radiometer (AVHRR) global land cover data with a spatial resolution of 1 km, which were generated by the University of Maryland (Hansen et al., 2000) and obtained from the website (http://daac.ornl.gov/cgi-bin/MODIS/GR_col5_1/mod_viz.html). The vegetation types were reclassified as needle-leaved forest (NDF), mixed forest (MF), broadleaved forest (BDF), woodland (WDL), wooded grassland (WG), shrubland (SHB), cropland (CRP), grassland (GRL) and other land cover types. The nearest-neighbor interpolation method was also performed to resample land cover data to a resolution of 1/12°. WGS-84 coordinate system was adopted for all datasets.

### Simple linear trend analysis

We adopted the ordinary least-squares (OLS) approach to determine the spatio-temporal change trend of the NDVI, precipitation and temperature at different growth periods, respectively (Piao et al., 2011b; Xia et al., 2018). The slope was calculated using the following formula:

(2) \begin{eqnarray*}Slope= \frac{n\times \sum _{j=1}^{n}j\times {k}_{j}- \left( \sum _{j=1}^{n}j \right) \left( \sum _{j=1}^{n}{k}_{j} \right) }{n\times \sum _{j=1}^{n}{j}^{2}-{ \left( \sum _{j=1}^{n}j \right) }^{2}} \end{eqnarray*}

where *n* represents the total number of years; *j* is the serial number from 1 to *n*; and k_*j*_ is the value of parameter (the NDVI, precipitation, temperature) of year *j*. If the slope greater than 0, which indicates an increasing trend, and if the slope less than 0, which shows a decreasing trend.

In addition, the significance level of change trend was assessed using the *t*-test, and the *p*-value was adopted to reflect the significance level. If the *p*-value was less than 0.05, and we considered that the change trend was significant.

### Correlation analysis

We applied correlation analysis to explore controlling factors of vegetation growth (Peng et al., 2011; Piao et al., 2011b). Pearson correlation coefficients were calculated among the NDVI and precipitation and temperature for the growing season, spring, summer and autumn, respectively.

For the in-deep understanding of the correlations between vegetation growth and climate factors, we analyzed the changes of correlation between the NDVI and climate factors in growing season, spring, summer and autumn. Previous research (Chen et al., 2015; Cong et al., 2017; Piao et al., 2014) suggested that relationship between the NDVI and climatic factors was influenced by the general changes of background climate over a fifteen-year period. We then performed a Pearson correlation analysis with a 15-year moving window to calculate the correlation coefficients between the NDVI and climates variables (Cong et al., 2017). For instance, the correlation coefficient of the year 1989 was calculated in the first 15-year window from 1982 to 1996. Then, to calculate the correlation coefficient of 1990, we moved forward one year to the second 15-year window (1983–1997). By analogy, until the last 15-year window, we calculated the correlation coefficient of the year 2008.

### Residual trend analysis

Climate and anthropogenic factors are closely related to vegetation growth in arid and semi-arid regions (Li, Liu & Fu, 2011; Zhou, Yamaguchi & Arjasakusuma, 2017; Funk & Brown, 2006). We adopted pixel-based residual trend analysis to distinguish the human-induced NDVI changes from the changes induced by climate factors. We first defined the residual as the difference between the satellite-derived NDVI and the predicted NDVI (Evans & Geerken, 2004; Zhou, Yamaguchi & Arjasakusuma, 2017). The equation is shown as follows (Liu & Liu, 2018).

(3) \begin{eqnarray*}NDVIr=NDVIs-NDVIc\end{eqnarray*}

where *NDVIr* is NDVI residuals; *NDVIs* is satellite-derived NDVI; and *NDVIc* is predicted NDVI values based on precipitation and temperature, which is calculated using the following formula (Liu & Liu, 2018; Li, Liu & Fu, 2011).

(4) \begin{eqnarray*}NDVIc={a}_{1}\mathrm{ \ast }tmp+{a}_{2}\ast pre\end{eqnarray*}

where *a*_1_ and *a*_2_ are the regression coefficients; and *tmp*, *pre* are the average temperature and precipitation for growing season from 1982–2015.

Then, simple linear trend analysis was used to calculate the changes of residuals at pixel scale (Zhou, Yamaguchi & Arjasakusuma, 2017). If there was a statistically significant decreasing trend, the degradation of vegetation was considered to be mainly induced by human activities. If there was a statistically significant increasing trend, human activities were considered to have a positive effect on vegetation growth. We calculated the NDVI residual trend and classified the change trend as significant increase, significant decrease and insignificant change.

## Results

### NDVI and climatic factors changes at temporal scales

### Temporal trend of NDVI and climate factors

**Figure 2:**
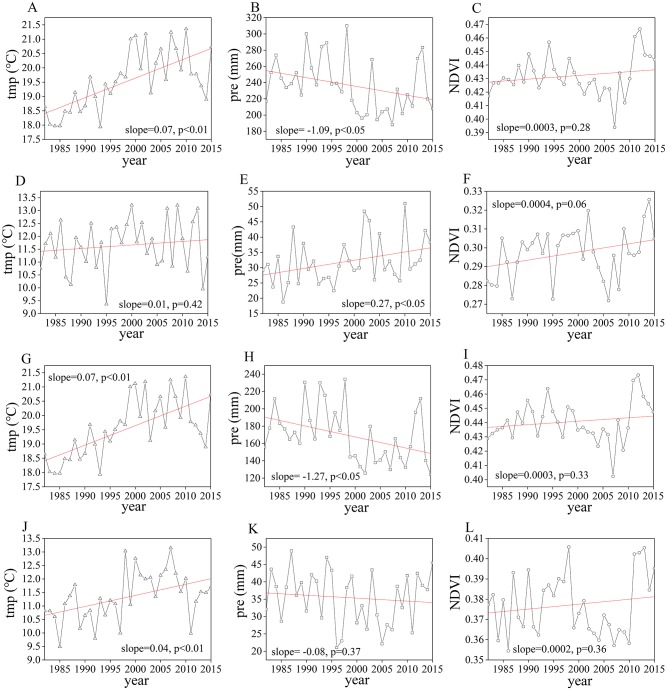
Interannual changes of temperature (tmp), precipitation (pre) and NDVI in growing season, spring, summer and autumn, respectively. Interannual changes of temperature (tmp) (A), precipitation (pre) (B) and NDVI (C) in growing season; interannual changes of temperature (tmp) (D), precipitation (pre) (E) and NDVI (F) in spring; interannual changes of temperature (tmp) (G), precipitation (pre) (H) and NDVI (I) in summer; interannual changes of temperature (tmp) (J), precipitation (pre) (K) and NDVI (L) in autumn.

As shown in Figs. 2A and 2B, in growing season, the Mongolian Plateau exhibited significantly drier and warmer trend over the past three decades (slope = − 1.09 mm/yr, *p* < 0.05; slope=0.07 °C/yr, *p* < 0.01). Vegetation growth showed a greening trend with the rate of 0.0003 per year in growing season (Fig. 2C), but the trend was insignificant (*p* = 0.28). For three seasons, spring temperature and precipitation (Figs. 2D and 2E) showed an increasing trend (slope = 0.01 °C/yr (*p* = 0.42) and 0.27 mm/yr (*p* < 0.05), respectively), and spring NDVI also had an increasing tendency (Fig. 2F), with the rate of 0.0004 per year (*p* = 0.06). Similar to the climate change of growing season, a warmer and drier trend was also found in summer (Figs. 2G and 2H: slope = 0.07 °C/yr (*p* < 0.01) and −1.27 mm/yr (*p* < 0.05), respectively) and autumn (Figs. 2J and 2K: slope = 0.04 °C/yr (*p* < 0.01) and −0.08 mm/yr (*p* = 0.37), respectively). NDVI of the two seasons (Figs. 2I and 2L) also showed an insignificant rate of increase at 0.0003 per year (*p* = 0.33) and 0.0002 per year (*p* = 0.36), respectively.

### Relationship between the interannual NDVI and climate factors

Table 1 shows the Pearson correlation coefficients between the NDVI and precipitation (R_NDV I_pre_) and temperature (R_NDV I_tmp_) for growing season, spring, summer and autumn. The NDVI during growing season and summer was significantly positively correlated with precipitation, with correlation coefficients of 0.54 and 0.50, respectively. The NDVI of the two growth periods showed an insignificant negative correlation with temperature, both with correlation coefficients of −0.14. The spring NDVI was positively correlated with temperature and precipitation, but only the correlation between the NDVI and temperature was significant. The autumn NDVI was insignificantly correlated with precipitation and temperature, with correlation coefficients of −0.007 and 0.19, respectively.

**Table 1:** Pearson correlation coefficients between the NDVI and climate factors. Pearson correlation coefficients between the NDVI and climate factors in growing season, spring, summer and autumn, respectively.

**Growth stage**	**Growing season**	**Spring**	**Summer**	**Autumn**
R_NDV I_pre_	0.54^**^	0.24	0.5^**^	−0.007
R_NDV I_tmp_	−0.14	0.44^*^	−0.14	0.19

**Notes.**

* and ** represent *p* < 0.05 and *p* < 0.01, respectively.

### Changes of Pearson correlation coefficients between interannual NDVI and climate factors

**Figure 3:**
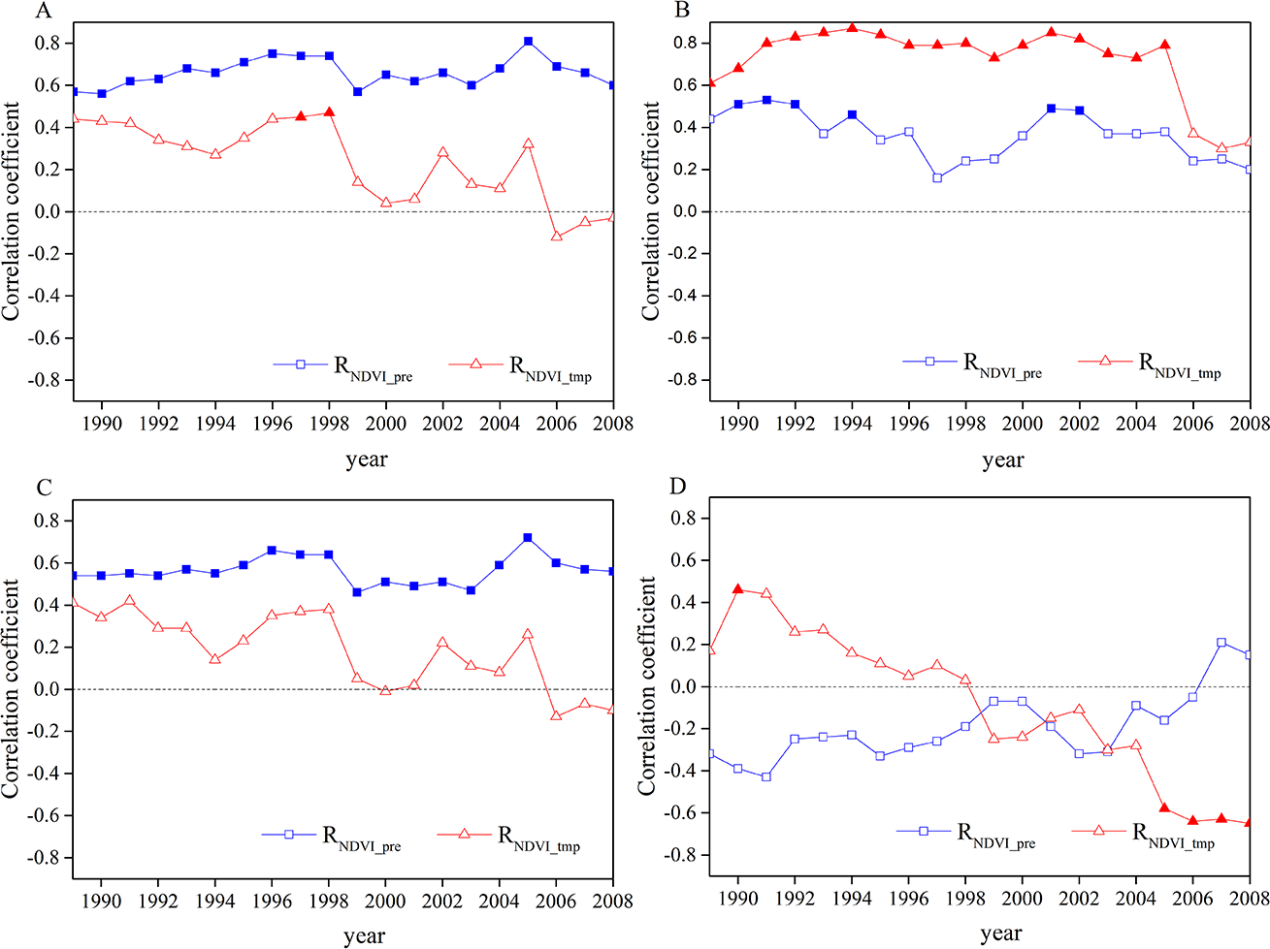
Changes of R_NDV I_pre_ and R_NDV I_tmp_ for (A) growing season, (B) spring, (C) summer and (D) autumn. The solid symbols represent a significant correlation with a 95% confidence level.

To further investigate the effects of climatic factors on vegetation growth over time, we analyzed the relationships among the NDVI and precipitation and temperature using the Pearson correlation method with a 15-year window. The growing season NDVI was significantly positively correlated with precipitation (Fig. 3A), and the correlation coefficients showed no obvious changes. The correlation coefficients between growing season NDVI and temperature (R_NDV I_tmp_) were positive before 2005, and R_NDV I_tmp_ was significant only in 1997 and 1998. The absolute values of R_NDV I_pre_ were all greater than R_NDV I_tmp_ values, indicating that growing season NDVI was more sensitive to precipitation than temperature. R_NDV I_pre_ and R_NDV I_tmp_ for summer (Fig. 3C) showed the same trends as growing season.

The spring NDVI was positively correlated with temperature from 1989–2008 (Fig. 3B), and R_NDV I_tmp_ for spring showed significant correlation with temperature, with the exception of 2006, 2007 and 2008. Though R_NDV I_pre_ for spring was also positive from 1988–2008, the values of R_NDV I_pre_ were all less than those of R_NDV I_tmp_, suggesting that temperature was the main factor influencing the spring NDVI changes. We found that values of R_NDV I_pre_ and R_NDV I_tmp_ during autumn (Fig. 3D) showed notable changes from 1989–2008. Besides, R_NDV I_pre_ of autumn showed an increasing tendency, whereas R_NDV I_tmp_ showed a decreasing trend.

### Spatial distributions of climatic factors and NDVI trends

#### Spatial distributions of climate factors

**Figure 4:**
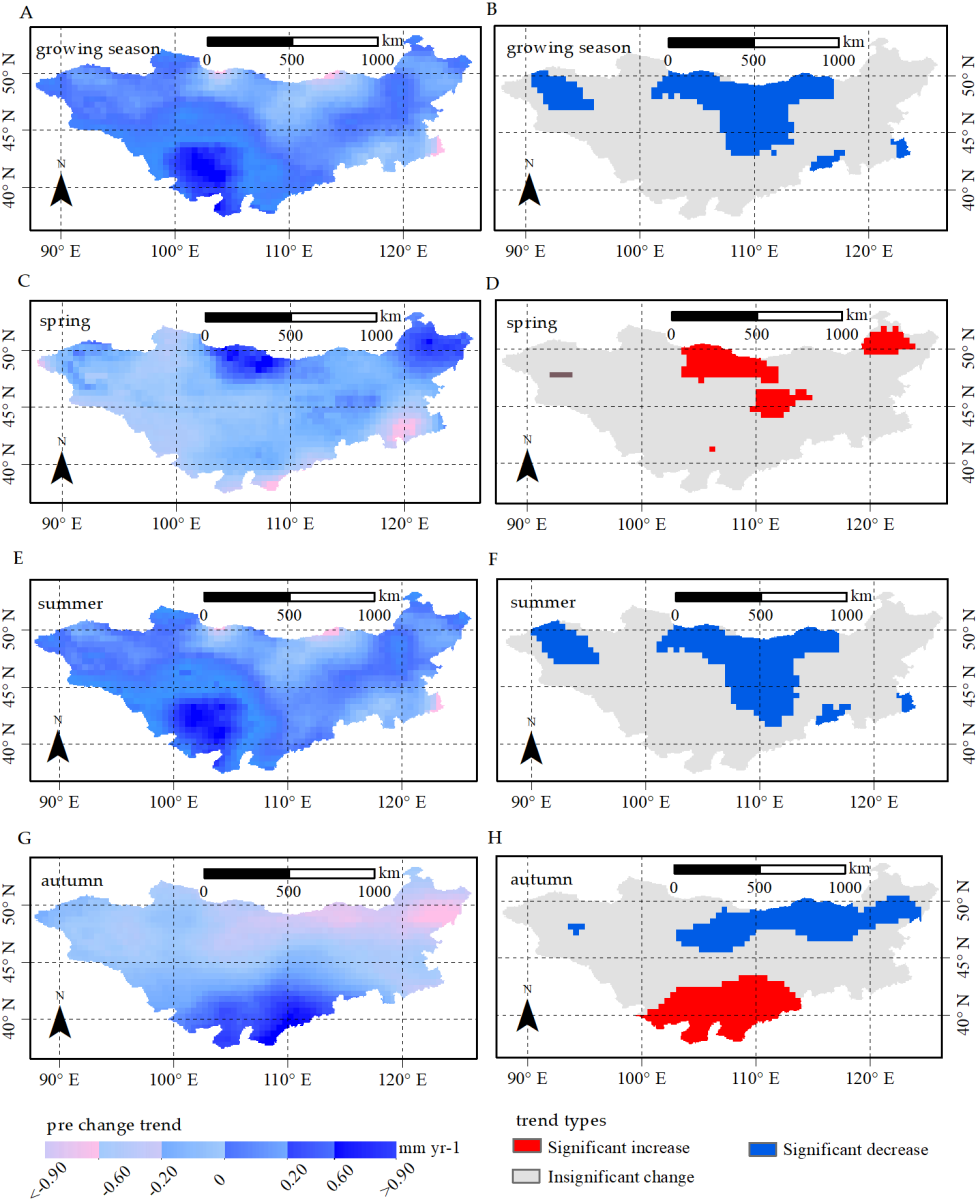
Change trend and change trend types for precipitation in growing season, spring, summer and autumn, respectively. Change trend for precipitation (pre) in (A) growing season, (C) spring, (E) summer and (G) autumn; *p*-values of pre change trend in (B) growing season, (D) spring, (F) summer and (H) autumn. The significant increases and significant decreases are represented by the *p*-value <0.05, and insignificant change has a *p*-value >0.05.

**Figure 5:**
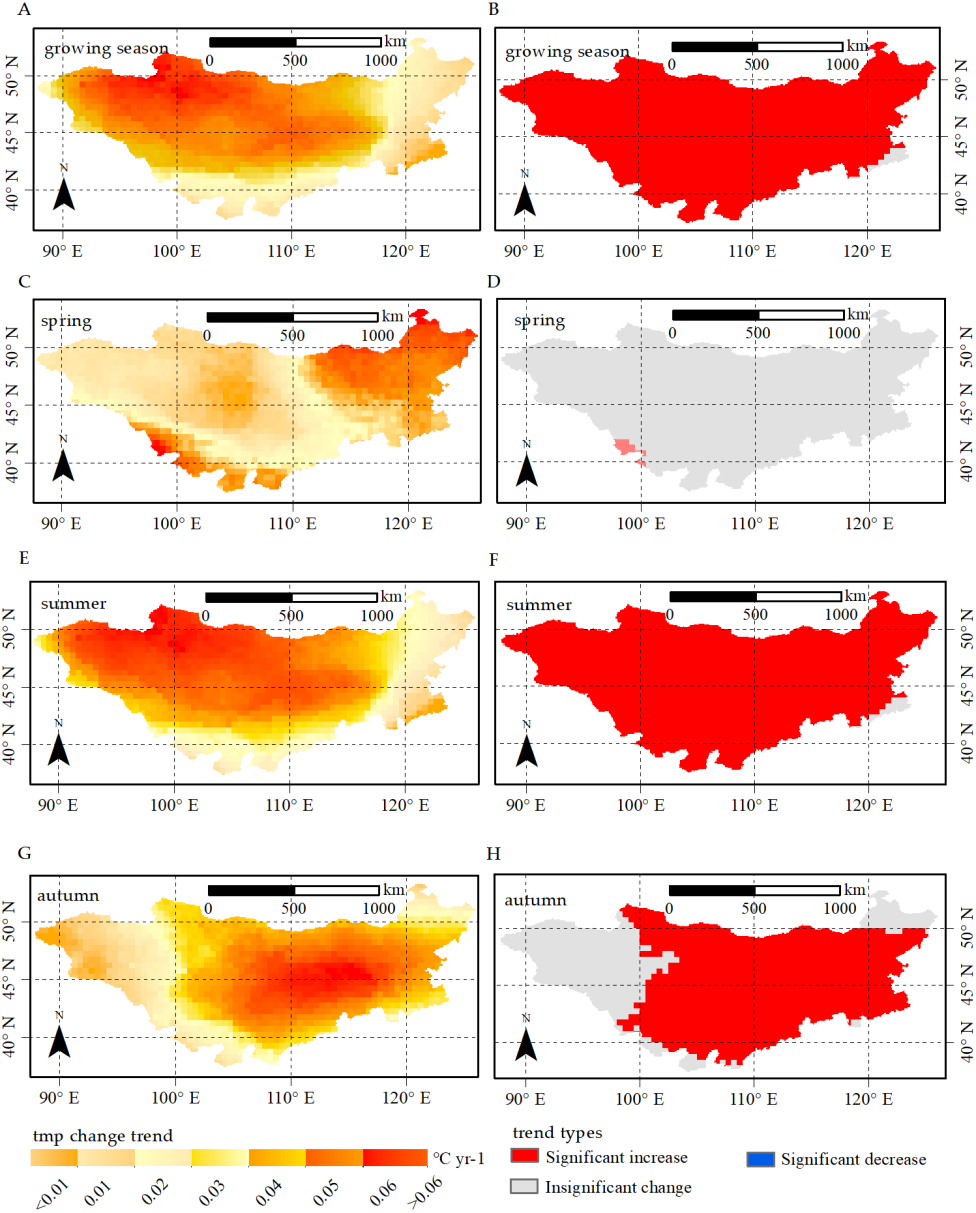
Change trend and change trend types for temperature in growing season, spring, summer and autumn, respectively. Change trend for temperature (tmp) in (A) growing season, (C) spring, (E) summer and (G) autumn; *p*-values of tmp change trend in (B) growing season, (D) spring, (F) summer and (H) autumn. The significant increases and significant decreases are represented by the *p*-value < 0.05, and insignificant change has a *p*-value > 0.05.

Figures 4 and 5 show the spatial distributions of precipitation and temperature trends from 1982 to 2015. We found a drier trend during the growing season, summer and autumn, with a decrease in precipitation for 85.9%, 89.5% and 71.1% of the whole area, respectively, primarily located in southeastern and northern parts of the plateau. Nevertheless, in spring, a wetter trend was observed in a large region (86.1%) in northern parts of the plateau, and we observed a significant increase in spring precipitation in the northern plateau, accounting for 11.6% of the entire region.

Furthermore, we found that temperature of growing season and summer significantly increased for nearly 99% of the plateau. In particular, the increasing rates of northwestern and central areas were larger than those of northeastern areas in the region. We also found large areas (88.4% and 99%) with increasing temperature in spring and autumn, with significant increases in 0.7% and 70.5% of the area, respectively. Overall, most areas in the Mongolian Plateau showed a warming trend over the past three decades.

#### Spatial distribution for NDVI trend

**Figure 6:**
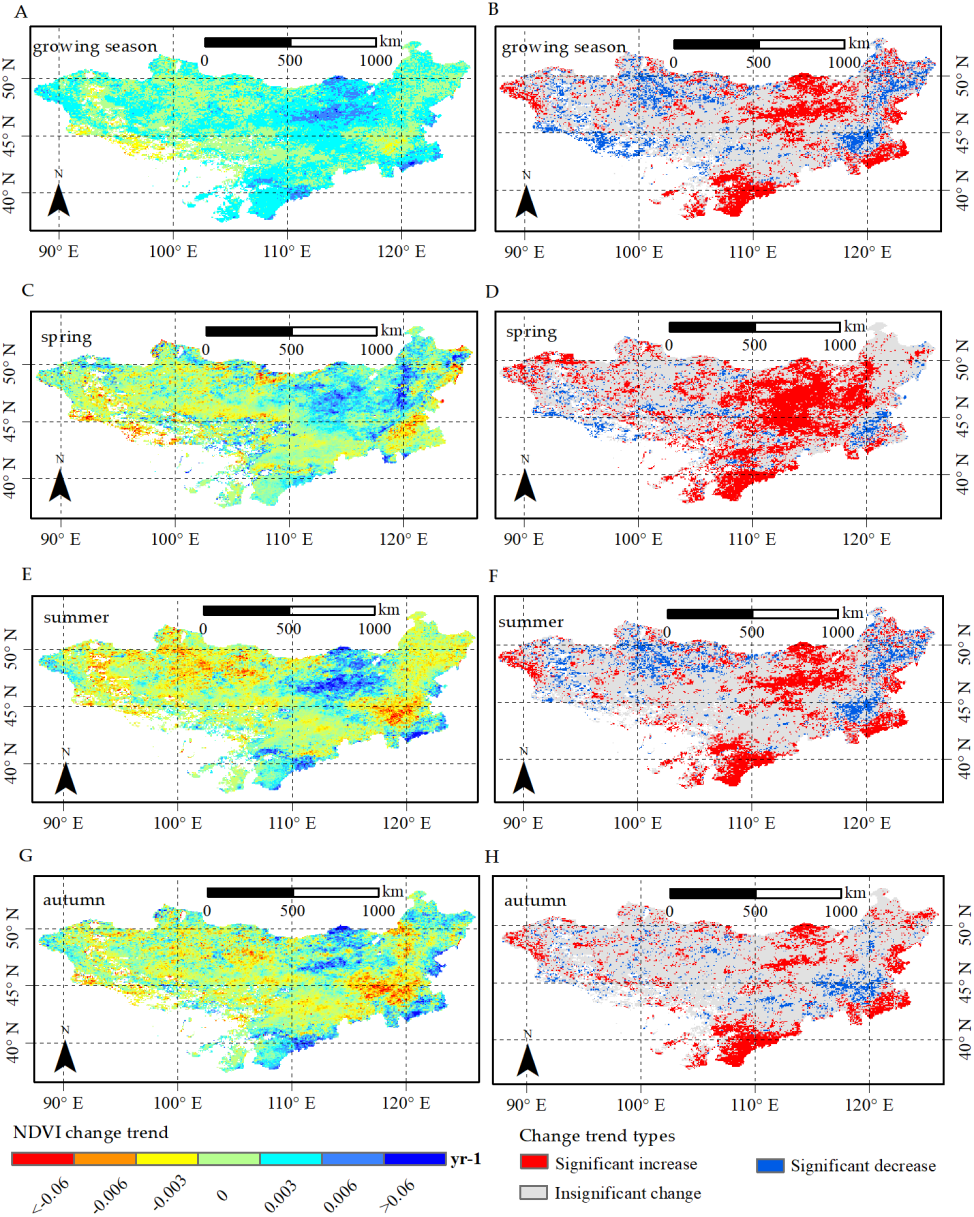
Change trend and change trend types for NDVI in growing season, spring, summer and autumn, respectively. Change trend for NDVI in (A) growing season, (C) spring, (E) summer and (G) autumn; *p*-values of NDVI change trend in (B) growing season, (D) spring, (F) summer and (H) autumn. The significant increases and significant decreases are represented by the *p*-value <0.05, and insignificant change has a *p*-value >0.05.

Pixel-based simple linear trend analysis was applied to explore the spatial patterns of NDVI changes for growing season, spring, summer and autumn. As shown in Fig. 6, the NDVI trend showed high spatial heterogeneity during 1982–2015. We found the growing season NDVI (Fig. 6A) decreased in 46.2% of the Mongolia Plateau, with a significant decline in 12.8% of total area. In particular, the decrease rates of southwestern plateau exceeded 0.003 per year, and the area were distributed in shrublands. Nevertheless, we also observed a large region (53.8%) with the increasing NDVI in growing season, and the increase was significant in 21.4% of the plateau. In particular, the increase rates of the NDVI at the southern and northern plateau regions covered by grasslands and shrublands were over 0.006 per year. For the seasonal NDVI, the regions with the increasing spring NDVI (Fig. 6C) accounted for approximately 71.1% of the total area. Furthermore, a large region (33.8% of the plateau) showed a significant increase trend, which was located in the southern and northern Mongolian Plateau and primarily distributed in shrublands and grasslands. In contrast, 6.1% of the plateau presented a significant decrease trend in spring NDVI, which was scattered in the east of the plateau and dominated by croplands. The spatial pattern of summer NDVI (Fig. 6E) change trend was similar to that of the growing season. In autumn (Fig. 6G), the regions with increasing NDVI accounted for about 60.2% of the plateau. Among them, 18.2% of the total area showed a significant increase trend, which was scattered in the southern and southeastern plateau primarily with shrublands and grasslands. Meanwhile, we found the NDVI in autumn significantly decreased in 5.9% of the plateau, and was primarily scattered in the east of the plateau covered by croplands.

Table 2 displays the change trends for different vegetation types. In the growing season, NDVI of grasslands showed significant increasing trend, but the NDVI of other vegetation types all showed insignificant change trends. In addition, we found the NDVI of all vegetation types exhibited an increasing tendency in spring and autumn. Moreover, grasslands had the largest increase rate (slope = 0.0001) in spring, and the increase rate was significant. In summer, the NDVI of all vegetation types showed similar trends as observed in growing season.

**Table 2:** NDVI change trend for different vegetation types and climate factors, and Pearson correlation coefficients between NDVI and climate factors for different vegetation types. NDVI change trend for different vegetation types and climate factors, and Pearson correlation coefficients between NDVI and climate factors for different vegetation types in growing season, spring, summer and autumn, respectively.

**Periods**	**Vegetation type**	**Change trend from 1982–2015**			**Pearson correlation coefficients**	
		**NDVI**	**pre**	**tmp**	R_NDV I_pre_	R_NDV I_tmp_
Growing season	BDF	−0.00005	−0.72	0.05^**^	0.05	−0.07
	CRP	0.00004	−0.81^*^	0.06^**^	0.51^**^	−0.12
	GRL	0.0001^*^	−0.74^*^	0.07^**^	0.54^**^	−0.14
	MF	−0.00009	−0.67	0.05^**^	−0.02	0.08
	NLF	−0.00006	−0.81^*^	0.06^**^	0.02	0.02
	SHB	0.00002	−0.35	0.07^**^	0.72^**^	−0.81
	WDL	−0.00003	−0.8^*^	0.06^**^	0.21	−0.15
	WG	−0.00007	−0.8^*^	0.06^**^	0.46^**^	−0.28
Spring	BDF	0.00001	0.49^*^	0.03	−0.11	0.54^**^
	CRP	0.00004	0.23	0.02	0.08	0.45^**^
	GRL	0.0001^**^	0.2	0.01	0.17	0.33^*^
	MF	0.00009	0.54^*^	0.03	−0.15	0.54^**^
	NLF	0.00007	0.53^**^	0.02	−0.12	0.54^**^
	SHB	0.00007^*^	0.12	0.01	−0.05	0.32
	WDL	0.00001	0.45^*^	0.02	−0.09	0.59^**^
	WG	0.00002	0.31^*^	0.01	0.03	0.56^**^
Summer	BDF	−0.00005	−0.73	0.05^**^	0.05	−0.07
	CRP	0.00003	−0.84^*^	0.05^**^	0.5^**^	−0.12
	GRL	0.0001	−0.79^*^	0.07^**^	0.52^**^	−0.14
	MF	−0.00007	0.67	0.04^**^	−0.03	0.07
	NLF	−0.00005	−0.81^*^	0.06^**^	0.01	0.01
	SHB	0.00008	−0.42^*^	0.07^**^	0.6^**^	−0.07
	WDL	−0.00003	−0.82^*^	0.06^**^	0.21	−0.15
	WG	−0.00008	−0.83^*^	0.07^**^	0.46^**^	−0.28
Autumn	BDF	0.0000002	−0.56^*^	0.03	−0.26	−0.09
	CRP	0.000005	−0.35^*^	0.05^**^	0.1	−0.25
	GRL	0.000007	−0.23	0.04^**^	0.08	−0.02
	MF	0.00009	−0.53	0.03^**^	−0.34^*^	−0.02
	NLF	0.000008	−0.50^*^	0.04	−0.3	−0.07
	SHB	0.00007	0.08	0.03^*^	−0.01	0.11
	WDL	0.000001	0.52^**^	0.04^*^	−0.17	−0.16
	WG	0.000006	−0.40^*^	0.04^**^	0.08	−0.23

**Notes.**

*and ** represent *p* < 0.05 and *p* < 0.01, respectively.

#### Spatial distribution of correlation between NDVI and climatic factors

**Figure 7:**
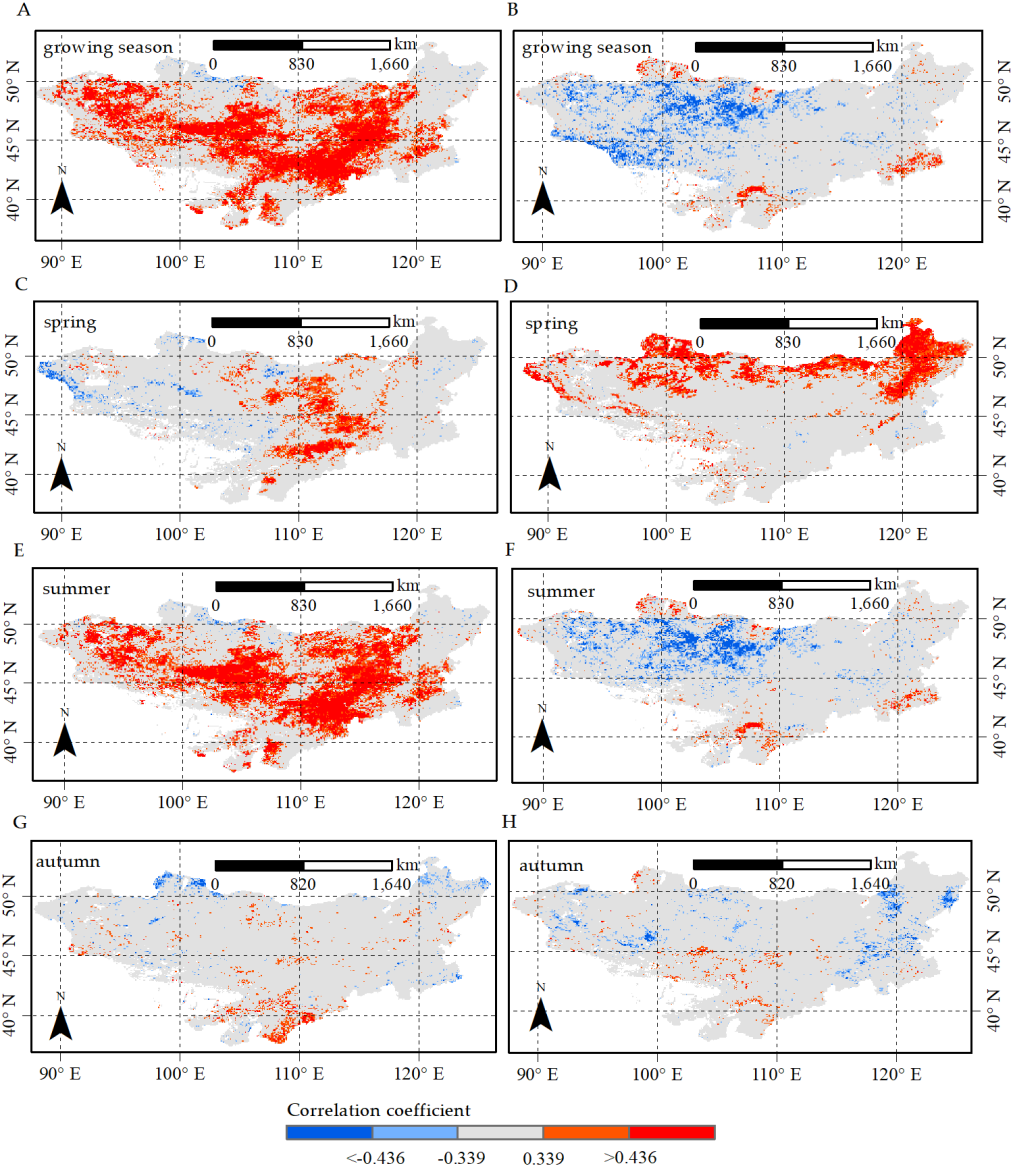
Correlation coefficient between NDVI and precipitation (pre) and temperature (tmp) in growing season, spring, summer and autumn, respectively. Correlation coefficient between NDVI and precipitation (pre) in (A) growing season, (C) spring, (E) summer and (G) autumn; correlation coefficient between NDVI and temperature (tmp) in (B) growing season, (D) spring, (F) summer and (H) autumn. The red represents that NDVI is significant positively correlated with climate factors; the blue represents that NDVI is significantly negatively correlated with climate factors; the light gray represents that NDVI is insignificantly correlated with climate factors.

We analyzed relationships among NDVI and precipitation and temperature at pixel level using the Pearson correlation method. As shown in Fig. 7A, a significant positive correlation between the NDVI and precipitation in growing season and summer was found in 41.2% and 38.4% of the plateau, respectively. Additionally, the regions were primarily located in the northwestern, central and southeastern Mongolian Plateau covered by grasslands, shrublands and croplands. Furthermore, we observed a significant negative correlation between the NDVI and temperature in growing season and summer (Fig. 7B), mainly scattered in the west of the plateau, which contained grasslands and shrublands, accounting for 13.1% and 11.6% of the total area, respectively. The correlation demonstrated that precipitation might be the main controlling factor for grasslands and shrublands growth in growing season and summer, and to a certain extent, temperature might limit the growth of grasslands and shrublands in the two growth periods.

Figure 7D showed the spring NDVI was significantly positively correlated with temperature (21.2%) in the northern plateau regions with mixed forests, broadleaved forests and needle-leaved forests. Additionally, we found NDVI was significantly positively correlated with precipitation (10.3%) in the central regions of the study area primarily with shrublands (Fig. 7C). The correlation suggested that temperature was the primary controlling factor for the growth of forests in spring. Moreover, the growth of shrubland in spring was mainly controlled by precipitation, even though precipitation generally increased during the spring (Fig. 4C). In autumn, we observed a significantly negative correlation between autumn NDVI and temperature in the eastern Mongolian Plateau mainly with croplands (Fig. 7H), accounting for 4.8% of the total area. The results suggested that temperature was the main limiting climate factor for autumn croplands growth.

We calculated the Pearson correlation coefficients between different vegetation types and precipitation and temperature (Table 2). The NDVI of all vegetation types, with the exception of mixed forests, was positively correlated with precipitation in growing season and summer. Furthermore, the NDVI of all vegetation types was negatively correlated with temperature during the two growth periods, except for mixed forests and needle-leaved forests. We also found the NDVI of all vegetation types was significantly and positively correlated with temperature in spring.

#### Spatial distribution of residual trends

**Figure 8:**
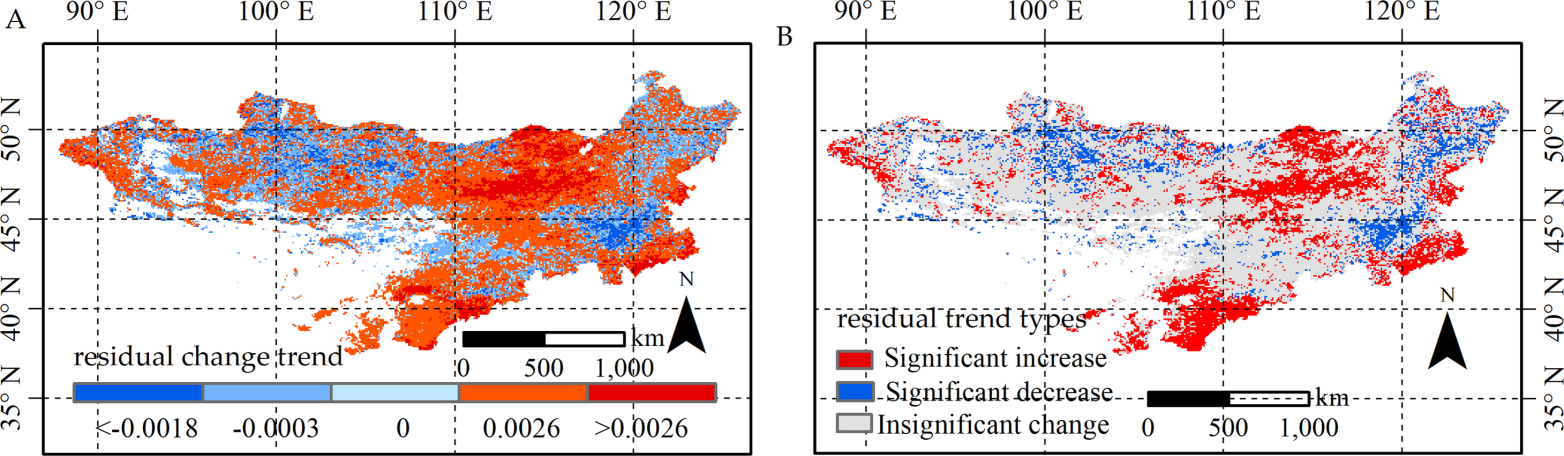
Change trend and change trend types for residual in the Mongolian Plateau. The spatial distribution of residual change trend (A) and its *p*-values (B). The significant increases and significant decreases are represented by the *p*-value <0.05, and insignificant change has a *p*-value >0.05.

Vegetation growth in the Mongolian Plateau was affected by both climate factors and human activities. Distinguishing the impacts of anthropogenic and climate factors played an important role in vegetation management. As shown in Fig. 8A, there were a large area (57.5%) showed an increasing residual trend, primarily located in the southeastern, central northern regions with grasslands, southern regions with shrublands, and the significant increase accounted for 23.6% of the plateau (Fig. 8B). We found the residuals decreased in 42.7% of the plateau, and the decrease was significant in 11.3% of the area, mainly scattered in the northern and eastern plateau, and cropland primarily distributed in these regions.

## Discussion

Over the past three decades, the Mongolian plateau showed a drier and warmer trend in growing season, which also corresponded to the fact of global warming (Guo et al., 2017; Piao et al., 2008). To some extent, increasing temperature can enhance vegetation productivity and extend the length of the growing season (Liu et al., 2017; Tsvetsinskaya et al., 2003). Consequently, the increasing trend of NDVI in the Mongolian Plateau may be the result of a warmer climate. This finding is consistent with previous research made by Wendurina et al. (2017) and Zhang et al. (2018). John et al. (2013) found extreme climatic events had a significant influence on vegetation growth. Compared with other years, the growing season NDVI was lower from 2000 to 2009, which may be a result of the high frequency of extreme drought during this period of time, and this finding was in line with previous studies made by John et al. (2013) and Tong et al. (2018). In our study, the plateau showed a warmer and wetter trend in spring, and the vegetation growth showed a tendency of greening. Appropriate increases of temperature and precipitation can promote photosynthesis rates, which was also found in the study of Liu et al. (2013).

Vegetation growth is greatly affected by climate factors in temperate regions. As shown in Fig. 1A, the spatial distribution of vegetation types across the Mongolian Plateau varied with spatial differences of climate. At the temporal scale, the growing season and summer NDVI were primarily dominated by precipitation, while the spring NDVI was more sensitive to temperature changes, which was in line with previous studies (Chen et al., 2015; Piao et al., 2011b). Spatially, the responses of NDVI for different vegetation types to climate factors were heterogeneous in growing season, spring, summer and autumn. Shrublands were mainly distributed in the southern and central regions of the study area, which were also the main distribution areas of desert in the Mongolian plateau (Figs. 1B and 1C). Its NDVI showed a positive correlation with precipitation in growing season and summer, even in spring. This result suggested that precipitation was the vital controlling factor for shrublands growth, and similar findings were also reported in Eckert et al. (2015) and Shao et al. (2018). Grasslands and croplands were distributed in the northern and eastern parts of the plateau, and we also observed that the NDVI of grasslands and croplands in these areas also showed a positive correlation with precipitation in growing season and summer. Broadleaved, mixed and needle-leaved forests were distributed in regions of the northern and northeastern plateau with sufficient amounts of precipitation (Fig. 1C), and temperature was observed to be the main controlling factor for forests growth in spring. The finding was consistent with Piao et al. (2011a).

In this study, we highlighted anthropogenic contributions to vegetation growth. Our results suggested that human activities promoted the growth of grasslands. This could be explained by the fact that policy implementation led to croplands abandonment and grazing exclusion in favor of grasslands restoration. For instance, IMAR has been benefiting from large-scale ecological restoration programs launched by China’s central and local governments since the late 1990s, such as Grain for Green Project, which has a positive impact on grassland growth in IMAR (Zhou, Yamaguchi & Arjasakusuma, 2017). Human-induced vegetation degradation regions were primarily located in the eastern and northern plateau covered by croplands. The results revealed that human activities (conversion of land use types, ‘Grain for Green’ project, etc.) led to the rapid decrease of croplands, and the finding was also reported by previous studies conducted by Ao et al. (2010), Mu et al. (2012) and Shao et al. (2018).

Our study provides preliminary findings that may contribute additional knowledge about vegetation growth and its responses to climate and anthropogenic factors in the Mongolian Plateau. Furthermore, our study may offer suggestions for policy makers towards sustainable land management and facilitate the sustainable development of the terrestrial ecosystems. Additionally, these basic findings imply that it would be better for future studies to combine ecological models to explore the associated effects on vegetation changes.

## Conclusions

The vegetation growth has been influenced by climate and human factors in the Mongolian Plateau, and understanding the relationships between vegetation growth and climate and anthropogenic factors are important for global change studies and land management. Using remote sensing data for 1982–2015, we showed that the Mongolian Plateau became drier and warmer over the past 30 years, and the growing season NDVI showed an insignificant increasing trend over the entire plateau. Furthermore, changes in NDVI of different vegetation types also varied in different growth periods. In growing season, most vegetation growth was positively correlated with precipitation. However, temperature was the main controlling factor for spring vegetation growth, especially for forests growth. Human activities promoted the growth of grasslands and shrublands, while croplands decreased greatly due to policy implementation. These findings help to better learn the relations between NDVI changes and climatic and human factors, and may offer suggestions for the sustainable management of the Mongolian Plateau. In addition, ecological models maybe considered to more accurately explore the associated effects on vegetation changes in the future.

## Additional Information and Declarations

### Data Availability

The following information was supplied regarding data availability: Data is available at: University of East Anglia Climatic Research Unit; Harris, I.C.; Jones, P.D. (2019): CRU TS4.03: Climatic Research Unit (CRU) Time-Series (TS) version 4.03 of high-resolution gridded data of month-by-month variation in climate (Jan. 1901- Dec. 2018). Centre for Environmental Data Analysis. http://catalogue.ceda.ac.uk/uuid/10d3e3640f004c578403419aac167d82. NDVI data is available at: https://ecocast.arc.nasa.gov/data/pub/gimms/3g.v1. Land cover data is available at: https://globalmaps.github.io/glcnmo.html.
